# Successful treatment of arrhythmia with β‐blocker and flecainide combination in pregnant patients with Andersen–Tawil syndrome: A case report and literature review

**DOI:** 10.1111/anec.12798

**Published:** 2020-09-21

**Authors:** Pongprueth Rujirachun, Apichaya Junyavoraluk, Manop Pithukpakorn, Bhoom Suktitipat, Arjbordin Winijkul

**Affiliations:** ^1^ Faculty of Medicine Siriraj Hospital Mahidol University Bangkok Thailand; ^2^ Division of Medical Genetics Department of Medicine Faculty of Medicine Siriraj Hospital Mahidol University Bangkok Thailand; ^3^ Siriraj Center of Research Excellence in Precision Medicine Faculty of Medicine Siriraj Hospital Mahidol University Bangkok Thailand; ^4^ Department of Biochemistry Faculty of Medicine Siriraj Hospital Mahidol University Bangkok Thailand; ^5^ Division of Cardiology Department of Medicine Faculty of Medicine Siriraj Hospital Mahidol University Bangkok Thailand

**Keywords:** Andersen–Tawil syndrome, case report, flecainide, pregnancy, β‐blocker

## Abstract

Andersen–Tawil syndrome (ATS) is a rare disorder characterized by a triad of ventricular arrhythmia (VA), dysmorphic features, and periodic paralysis. Due to the rarity of this condition, less is known about physiologic effect of pregnancy to ATS and arrhythmia. There is no established guideline for peripartum or postpartum treatment and prevention of arrhythmia in ATS; thus, the clinical management is challenging. We reported two *KCNJ2*‐associated ATS patients who got pregnant and underwent vaginal birth safely. Both individuals had VA, micrognathia without periodic paralysis. β‐blocker plus flecainide could be an effective treatment combination when monotherapy failed to control arrhythmia. VA of two pregnant patients with ATS could be controlled by either physiologic changes associated pregnancy or the combination treatment of β‐blocker and flecainide.

## 1 INTRODUCTION

Andersen–Tawil syndrome (ATS) is a rare autosomal dominant channelopathy characterized by clinical triad of ventricular arrhythmia (VA), periodic paralysis, and distinctive dysmorphic features.

Due to the rarity of this condition, less is known about physiologic effect of pregnancy to ATS and arrhythmia. There is no established guideline for prenatal or postpartum treatment and prevention of arrhythmia in ATS; thus, the clinical management is challenging (Roston et al., 2020; Subbiah et al., 2008). Here, we reported two pregnant ATS patients whose VA could be controlled by antiarrhythmic drug combination.

## 2 CASE REPORT

### 2.1 Case #1

**Figure 1:**
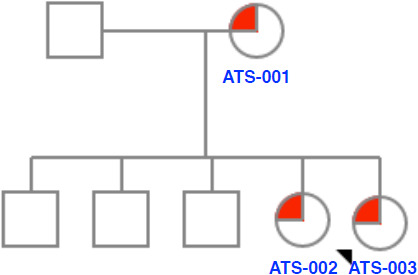
Family pedigree. The proband (ATS‐003) is indicated by the arrow. Family members with Andersen–Tawil syndrome (ATS) are illustrated as a red color (ATS‐001 and ATS‐002). Unaffected family members are illustrated as open symbols. A circle represents a female, and a square represents a male

**Figure 2:**
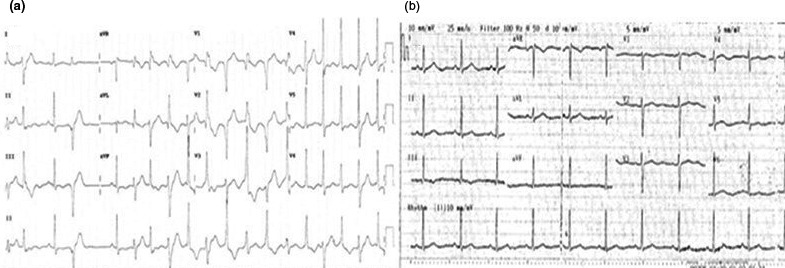
Twelve‐lead electrocardiography (ECG) demonstrating (a) polymorphic VT and (b) normal sinus rhythm

A 32‐year‐old Thai woman (Figure 1, ATS‐003) came for a medical advice on pregnancy planning in 2014. She had a history of abnormal heart rhythm found at general checkup since childhood. She was asymptomatic and grew up normally. The patient had one sister (ATS‐002) and three brothers. Her mother (ATS‐001) and sister experienced a similar history of abnormal heart rhythm. Both of them were also asymptomatic. All of her brothers were not affected. There was no history of sudden unexpected death in the family. She had another postmarital health checkup, and polymorphic premature ventricular contractions (PVC) with a short run of nonsustained polymorphic ventricular tachycardia (PMVT) were uncovered by electrocardiography (ECG) (Figure 2a). Further cardiac investigation by magnetic resonance imaging together with transthoracic echocardiography (TTE) showed left ventricular ejection fraction (LVEF) 25.1%, mild mitral valve regurgitation, mild tricuspid regurgitation, and no subendocardial scar. Only micrognathia was noted during physical examination without other dysmorphic features. Clinical diagnosis of Andersen–Tawil syndrome was suspected.

Genetic testing identified heterozygous likely pathogenic variant in *KCNJ2* c.557C > G (p.Pro186Arg) in all affected members (Figure 1). Treatment plan had been discussed between healthcare team and the patient. Oral metoprolol was given. The medication dose was gradually increased to 100 mg daily with no significant change of arrhythmias by periodic ECG monitoring. The patient decided to discontinue metoprolol after she became pregnant.

Her pregnancy was uneventful, and cardiac evaluation was done regularly. The ventricular ectopy became less frequent (Figure 2b). TTE at gestational age of 24 weeks showed mild reduction in left ventricular systolic function with left ventricular ejection fraction (LVEF) at 45%–50%. The patient was admitted to the hospital due to labor pain at 38 weeks of gestation. Continuous cardiac and fetal monitoring was instituted. Maternal ventricular ectopy was infrequent. Emergency cesarean delivery was done under spinal anesthesia due to irregular fetal heartbeats. There was no intrapartum and immediate postpartum complication. The patient was then transferred to cardiac care unit (CCU) for postoperative observation.

During CCU stay, she developed persistent PMVT. Metoprolol was resumed, and 100 mg of flecainide daily was added to successfully suppress the arrhythmia. The patient continued to do well until now at 5 months postpartum with occasional ectopic rhythm without ventricular tachycardia (VT).

### 2.2 Case #2

**Figure 3:**
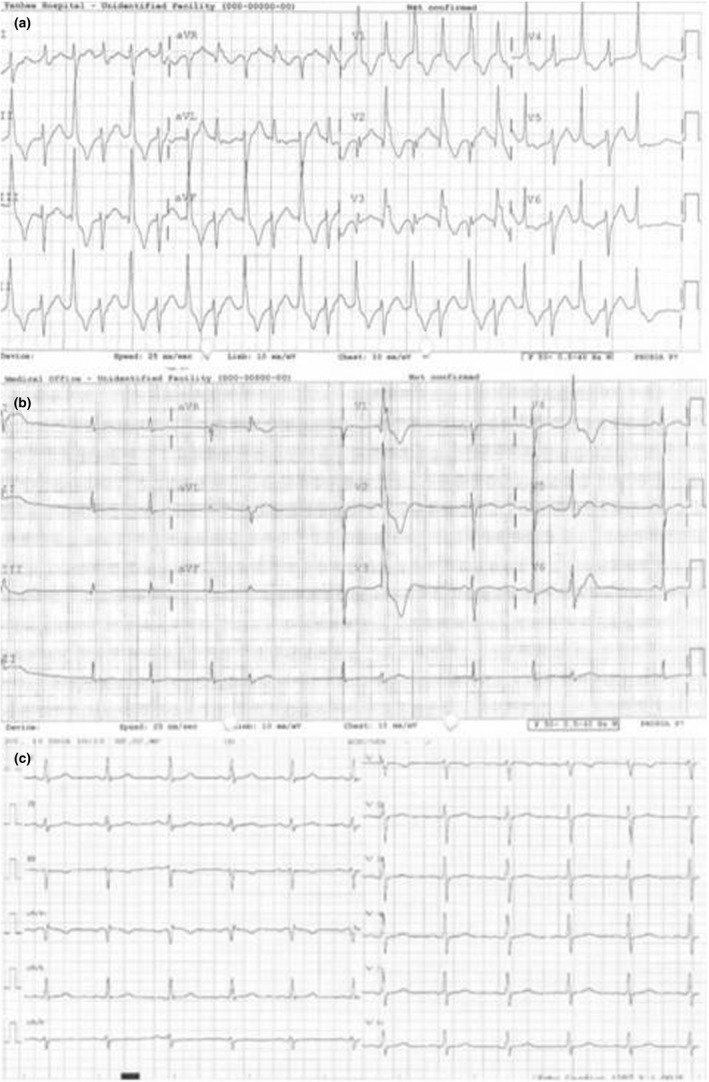
Twelve‐lead electrocardiography (ECG) demonstrating (a) incessant bidirectional VT, (b) sinus rhythm with prominent U wave and occasional premature ventricular contractions (PVCs), and (c) normal sinus rhythm

A 33‐year‐old woman (ATS‐002), sister of case #1 (ATS‐003), was also invited to our hospital for ATS evaluation. She was asymptomatic. Frequent bidirectional VT was noted on ECG (Figure 3a). TTE revealed systolic function at lower limits of normal with LVEF at 52%. Metoprolol was administered and gradually increased to 100 mg daily but her VA was not significantly reduced. One hundred mg of flecainide per day was then added, and cardiac monitoring showed ventricular ectopy becoming less frequent without VT (Figure 3b). Four months after treatment combination, 24‐hr Holter monitoring showed sinus rhythm with prominent U wave and occasional ventricular ectopy, and good biventricular systolic function with trivial mitral valve regurgitation was observed by TTE.

Similar to her sister, the patient decided to undergo her pregnancy without medication after extensive discussion with healthcare team. Her pregnancy was uneventful, and she delivered her child successfully. VT was noticed during postpartum monitoring; therefore, flecainide and metoprolol combination was resumed. Cardiac arrhythmia remained suppressed at 3‐month follow‐up visit (Figure 3c).

## 3 DISCUSSION

Majority of ATS individuals are caused by heterozygous mutations in *KCNJ2* which encodes for the α‐subunit of Kir2.1, an inward rectifier potassium channel responsible for late phase of cardiac repolarization. Classic ECG abnormalities include QTc prolongation and prominent U wave. Various types of VA, including bidirectional VT and PMVT, are prevalent and could be identified in more than 80% of the patients, though sudden cardiac death does not commonly occur (Fox et al., 2008). Current management strategy of ATS is mainly focused on suppression of ventricular ectopy and prevents tachycardia‐induced cardiomyopathy (Pellizzón et al. 2008).

These two *KCNJ2* [c.557C > G (p.Pro186Arg)]‐associated ATS patients who got pregnant and underwent vaginal birth safely without any medications were first reported. Both individuals had VA, micrognathia without periodic paralysis. β‐blocker plus flecainide were shown to be effective when β‐blocker alone could not suppress their arrhythmias.

Though the mechanism remains unknown, physiologic changes associated with pregnancy could have significant impact on cardiac conduction in patients with ATS. QTc interval is hormonally regulated, and increased estradiol is associated with shorter QTc interval via enhanced membrane trafficking of *KCNH2* (Anneken et al., 2016). One reported case also showed marked reduction in ventricular ectopy during pregnancy (Subbiah et al., 2008).

β‐blockers are considered mainstay therapy for inherited long QT syndromes, though β‐blocker‐resistant cases do occur (Moss et al., 2000; Roston et al., 2020). Flecainide is a fast‐inward sodium channel blocker with pleiotropic effects to reduce the oscillatory potentials. It can also terminate VT by modulating calcium dynamic via inhibiting reverse‐mode sodium–calcium exchanger or ryanodine receptor 2 and directly increasing Kir2.1 current with no significant impact on left ventricular function (Brembilla‐perrot et al., 1987). The medication was shown to be effective on reducing VA burden in multiple ATS patients (Fox et al., 2008; Pellizzón et al. 2008; Van Ert et al., 2016; Hayashi et al., 2014; Bökenkamp et al., 2007; Janson et al., 2014; Fernández et al., 2018) (Table 1). The synergistic effect of β‐blockers and flecainide might favor a combination treatment in ATS.

**Table 1:** Reported cases with success of Flecainide treatment in patients with ATS

Authors	Age/Sex	Clinical characteristics			Mutations of *KCNJ2* gene	Treatment	Result
		Dysmorphic features	Arrythmia	Periodic paralysis			
This case (2019)	32/F	Micrognathia	PMVT, NSPVT, polymorphic PVCs	N	c.557C > G (p.Pro186Arg), c.436G > C (p.Gly146Arg)	Flecainide 100 mg/day Metoprolol 100 mg/day	Improved
	33/F	Micrognathia	IBVT, bigeminy PVCs	N	c.557C > G (p.Pro186Arg), c.436G > C (p.Gly146Arg)	Flecainide 100 mg/day Metoprolol 100 mg/day	Improved
Fernándezet al. (2018)	25/M	Clinodactyly of 4th finger on right hand, 4th–5th fingers on left hand, and subtle facial dysmorphism (small jaw, low‐set ears, broad forehead, bulbous nose)	IBVT, bigeminy PVCs	Y	c.914C > T (p.Thr305Ala)	Flecainide 100 mg/12 hr Bisoprolol 5 mg/day Acetazolamide 250 mg/day	18‐month follow‐up, the patient remained free of palpitations and presented a low PVC burden under Holter monitoring.
Van Ert et al. (2016)	19/F	Hypertelorism, micrognathia, and low‐set ears	Polymorphic PVCs, bigeminy PVCs, PMVT, BVT	Y	c.653G > T (p.R218L)	Flecainide 100 mg/day Atenolol 25 mg/day	Resolution of symptoms. Repeat Holter monitoring showed that PVC burden decreased to < 1% with complete suppression of PMVT and BVT. Asymptomatic on stable antiarrhythmic therapy for 2 years.
Janson et al. (2014)	15/M	Micrognathia, wide‐space eyes, and clinodactyly of the 5th digit	BVT	—	p.Arg218Trp	Verapamil 4 mg kg^−1^ day^−1^ Flecainide 3–4 mg kg^−1^ day^−1^	Improvement of ectopy burden and VT suppression was sustained over 3 months of follow‐up.
Hayashi et al. (2014)	22/M	NR	Couplet, triplet PVCs, NSVT	Y	c.200G > A (p.R67Q)	Flecainide 100 mg/day Atenolol 50 mg/day	1 month after, PVCs significantly are reduced by 12‐lead ECG recording, treadmill exercise test, and 24‐hr ambulatory ECG recording and T‐wave alternan was almost diminished with shortening of QT interval during treadmill exercise test.
Fox et al. (2008)	54/M	Short stature (167 cm), broad forehead with associated hypotelorism	Polymorphic PVCs, BVT, NSPVT	Y	c.1132G > A (p.V302M)	Bisoprolol 5 mg/day conjunction with oral K + supplementation then switched to flecainide 200 mg/day	Holter monitoring and ETT, 2 weeks after the initiation of oral flecainide, showed dramatic reduction in all forms of VA (PVCs, couplets, and NSVT) Significant reduction in dyspnea during exercise and a 38% increase exercise capacity.
Pellizzón et al. (2008)	16/F	—	BVT	Y	p.R67W	Flecainide 200 mg/day 3 years then flecainide 300 mg/day	Effective in controlling BVT and reversing tachycardia‐induced cardiomyopathy.
Bökenkamp et al. (2007)	3/F	Broad forehead, hypertelorism, a small mandible, and clinodactyly	Polymorphic PVCs, PMVT, TdP tachycardia, VF	N	R218W	Intravenous flecainide 0.5 mg/kg Verapamil 0.1 mg/kg Verapamil 4 mg kg^−1^ day^−1^ Flecainide 4 mg kg^−1^ day^−1^ ICD	The patient had one appropriate ICD shock for TdP tachycardia during 3 years. During a total follow‐up to 6.5 years, ICD monitoring and repeat ECG and Holter recordings still show frequent polymorphic PVCs but no more runs of VT.
	6/M	Broad forehead, hypertelorism, and a small mandible	TdP tachycardia	Y	R218W	Flecainide 4 mg kg^−1^ day^−1^	Remained free of symptomatic arrhythmia during 5 years of follow‐up.

Abbreviations: BVT, bidirectional ventricular tachycardia; ECG, electrocardiogram; ETT, exercise tolerance testing; IBVT, incessant bidirectional ventricular tachycardia; ICD, implantable cardioverter defibrillator; N, no; NR, not reported; NSPVT, nonsustained polymorphic ventricular tachycardia; PMVT, polymorphic ventricular tachycardia; PVCs, premature ventricular contractions; TdP, torsade de pointes; VA, ventricular arrhythmia; VF, ventricular fibrillation; VT, ventricular tachycardia; Y, yes.

In concordance with previously reported cases, the combination of flecainide and β‐blocker was useful in patients with ATS when β‐blocker monotherapy failed to control cardiac arrhythmias.

## 4 CONCLUSIONS

We reported 2 ATS patients who underwent an uneventful pregnancy and childbirth and had effective control of ventricular arrhythmias with combination treatment of β‐blocker and flecainide.

## CONFLICT OF INTEREST

The authors have declared that no conflict of interests exist.

## AUTHOR CONTRIBUTIONS

Conceived and designed the experiments: PR, MP, BS, AW.

Performed the experiments: PR, MP, BS, AW.

Analyzed the data: PR, MP, BS, AW.

Contributed reagents/materials/analysis tools: BS AW.

Prepared figures and/or tables: PR, AJ.

Authored or reviewed drafts of the article: PR, AJ, MP, BS, AW.

Approved the final draft: PR, AJ, MP, BS, AW.

## ETHICS APPROVAL

The study protocol was approved by the Siriraj Institutional Review Board (Protocol number 197/2561).

## CONSENT FOR PUBLICATION

Verbal and written consent for publication was obtained from the patient.
